# Electroacupuncture Improves the Learning and Memory by Modulating Hippocampal Glucose Metabolism through IGF1/IGF1R Signaling in Alzheimer's Disease

**DOI:** 10.1002/advs.202514241

**Published:** 2026-05-23

**Authors:** Shengxiang Liang, Qingqing Zhang, Xiuxiu Wang, Lixin Peng, Xinru Ji, Jiawei Wang, Yusi Zhang, Jiayang Huang, Junchao Yang, Shiqi Zhan, Yiping Xiao, Weilin Liu, Lidian Chen

**Affiliations:** ^1^ National‐Local Joint Engineering Research Center of Rehabilitation Medicine Technology Fujian University of Traditional Chinese Medicine Fuzhou China; ^2^ Rehabilitation Industry Institute Fujian University of Traditional Chinese Medicine Fuzhou China; ^3^ Fujian Key Laboratory of Cognitive Rehabilitation Affiliated Rehabilitation Hospital of Fujian University of Traditional Chinese Medicine Fuzhou China; ^4^ College of Rehabilitation Medicine Fujian University of Traditional Chinese Medicine Fuzhou China

**Keywords:** alzheimer's disease, brain glucose metabolic network, electroacupuncture, hippocampus, IGF1/IGF1R signaling pathway, learning and memory

## Abstract

Alzheimer's disease (AD) is characterized by progressive cognitive decline and cerebral glucose hypometabolism. Emerging evidence suggests that modulating brain glucose metabolism represents a promising therapeutic strategy for AD. Here, we demonstrate that electroacupuncture (EA) improves cognitive function in 5×FAD mice by enhancing glucose metabolism in the hippocampus. EA treatment significantly attenuated learning and memory deficits and reduced β‐amyloid (Aβ) deposition in 5×FAD mice. Further analysis revealed that these improvements were associated with enhanced hippocampal glucose metabolism and optimized information processing in the brain metabolic network. In addition, EA specifically activated the IGF1/IGF1R signaling pathway in the hippocampus, which promoted membrane translocation of GLUT3 and consequently enhanced neuronal glucose uptake. The glucose metabolic enhancement boosted tricarboxylic acid cycle activity and improved synaptic plasticity. Our findings establish a novel mechanism by which EA improves the learning and memory through the IGF1/IGF1R pathway, providing both theoretic and experimental support for the clinical application of EA in AD treatment.

## Graphical abstract

Electroacupuncture (EA) ameliorates learning and memory function in 5×FAD mice by regulating the brain glucose metabolic network. This neuroprotective effect is closely related to enhancing neuronal energy utilization via the IGF1/IGF1R signaling pathway. These findings offer new insights into non‐pharmacological strategies for glucose metabolism in Alzheimer's disease.

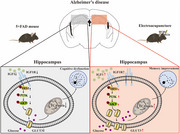

## 1 Introduction

Alzheimer's disease (AD) is a progressive neurodegenerative disorder which companied with learning and memory dysfunction [1, 2, 3]. It affects over 55 million people globally and causes heavy burdens for families and societies worldwide [4]. Reduced glucose metabolism in the hippocampus and temporoparietal lobe, as detected, is one of the hallmark features in AD [5, 6]. Brain glucose utilization in early AD patients is 12% lower than that of healthy peers and is significantly and positively correlated with cognitive score decline [7]. Several studies have shown that regulating brain energy metabolism is a promising strategy for improving the learning and memory in AD [8, 9, 10].

As a traditional Chinese medicine (TCM) therapy, acupuncture is regarded as an efficient strategy for AD due to its remarkable efficacy and fewer side effects [11, 12]. It has also been shown that acupuncture could activate hippocampal neural activity and enhance the connectivities in brain network, which significantly contributes to the memory function improvement of AD [13]. Besides, abnormal glucose metabolism in AD disrupts the neuronal energy supply, which significantly reduces the efficiency of synaptic information transmission. Ultimately, it dissociates the functional connectivity network in the whole brain [14, 15, 16]. In our previous study, we found that electroacupuncture (EA) could ameliorate cognitive decline in AD mice through upregulating GLUT3 expression in hippocampal neurons and promoting glucose uptake [17].

Recent researches have shown that the insulin‐like growth factor 1/insulin‐like growth factor 1 receptor (IGF1/IGF1R) signaling pathway is essential for neuronal glucose uptake and energy supply in AD [18, 19]. IGF1R is widely expressed in the hippocampus. The IGF1/IGF1R signaling pathway promotes the translocation of intracellular GLUT3 to the plasma membrane for glucose metabolism [20]. Meanwhile, the IGF1/IGF1R signaling pathway is closely associated with synaptic plasticity and impacts cerebral cellular survival by affecting mitochondrial function [19]. It has been found that acupuncture plays a role in upregulating IGF1 expression in the hippocampus [21]. However, it still remains unclear how EA modulates the IGF1/IGF1R signaling pathway to enhance glucose metabolism and improve cognitive function in AD.

In this study, we innovatively integrated PET imaging and metabolic network analysis to elucidate how EA improves the learning and memory in AD mice. We found that the neuroprotective effects of EA are associated with the activation of the IGF1/IGF1R signaling pathway. By driving the IGF1R‐mediated translocation of GLUT3 from the cytosol to the plasma membrane, EA enhances hippocampal glucose metabolism and synaptic plasticity. This molecular cascade subsequently strengthens whole‐brain metabolic network integration, which is essential for ameliorating the learning and memory deficits in 5×FAD mice. Our research not only provides important evidence for the neurobiological mechanism of acupuncture in treating AD, but also a new therapeutic insight for clinical intervention in cognitive disorders.

## 2 Results

### 2.1 EA Ameliorated Cognitive Impairments in AD Mice

**FIGURE 1:**
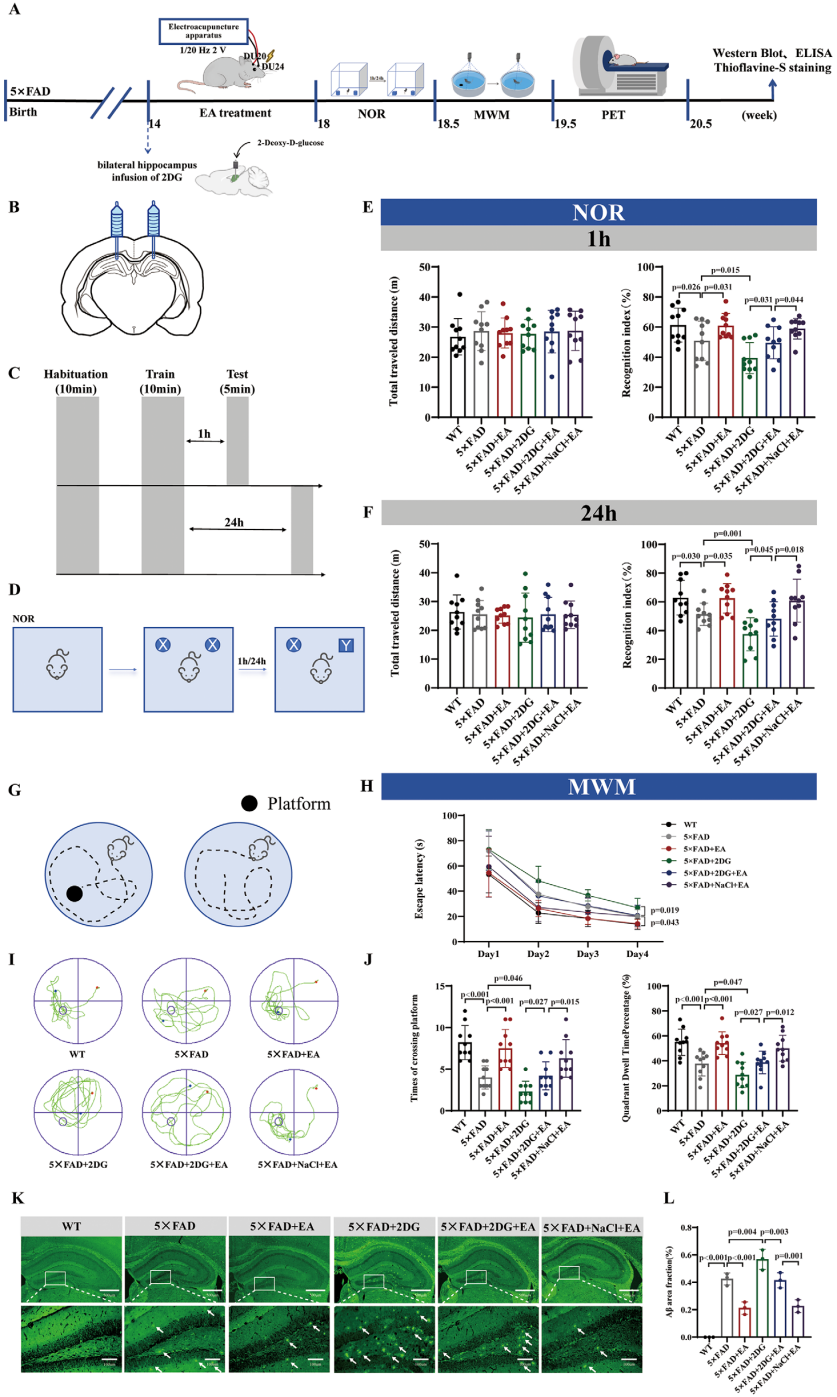
EA improved the learning and memory in 5×FAD mice. (A) Experimental procedures. (B) Schematic diagram depicting mice received 2DG injection in the bilateral hippocampus. (C,D) Schematic diagram of the NOR test. (E,F) Motor ability was comparable across all groups (E left; F left), and recognition index was assessed by the NOR test at 1 h (E right) and 24 h (F right) after training (*n* = 10 mice/group). (G) Schematic diagram of the MWM test. (H) Time spent before reaching the hidden platform in the MWM. (I) Representative swimming traces of 6 groups during the probe trial. (J) Number of entries into the platform (left) and time spent in the target quadrant (right) during the probe trial (*n* = 10 mice/group). (K) Representative images of Aβ (scale bars, 500 µm, 100 µm). (L) Quantification of Aβ area in the hippocampus (*n* = 3 mice/group, each n represents the average of three technical replicates). All data were shown as mean ± SEM. *P* values were indicated in the figure. Statistical analysis was performed using one‐way ANOVA with LSD comparisons (E, F, H, J, and L). MWM: Morris water maze; NOR:  Novel Object Recognition.

In this study, we systematically performed the EA intervention at “DU20” and “DU24” acupoints for 4 weeks in 5×FAD mice (Figure 1A). Besides, 2DG (2‐Deoxyglucose glucose metabolism inhibitor) or saline was bilaterally injected into the hippocampus through pre‐implanted guide cannulas prior to EA intervention (Figure 1B). Cognitive function assessment showed that the EA significantly improved the cognition of AD mice. In the new object recognition (NOR) experiment, the 5×FAD+EA group significantly increased the exploration time of new objects. And there was no difference in the moving distance among the six groups, which ruled out the interference of motor function (Figure 1C–F). Morris water maze (MWM) test further confirmed that the EA significantly shortened the escape latency, increased the number of platform traversals, and improved the proportion of time spent in the target quadrant, whereas 2DG suppressed these improvements. The above results confirmed the critical role of glucose metabolism (Figure 1G–J). Simultaneously, we also observed the effect of EA on the pathological deposition of Aβ in the hippocampal region. Thioflavin S staining revealed that EA significantly reduced Aβ pathological deposition of the hippocampus in 5×FAD mice, while these effects were suppressed by 2DG (Figure 1K,L). These results demonstrated that EA significantly improved the learning and memory in 5×FAD mice, as further evidenced by the fact that 2DG administration abolished these beneficial effects.

### 2.2 EA Improved the Brain Glucose Metabolic Network in AD Mice

**FIGURE 2:**
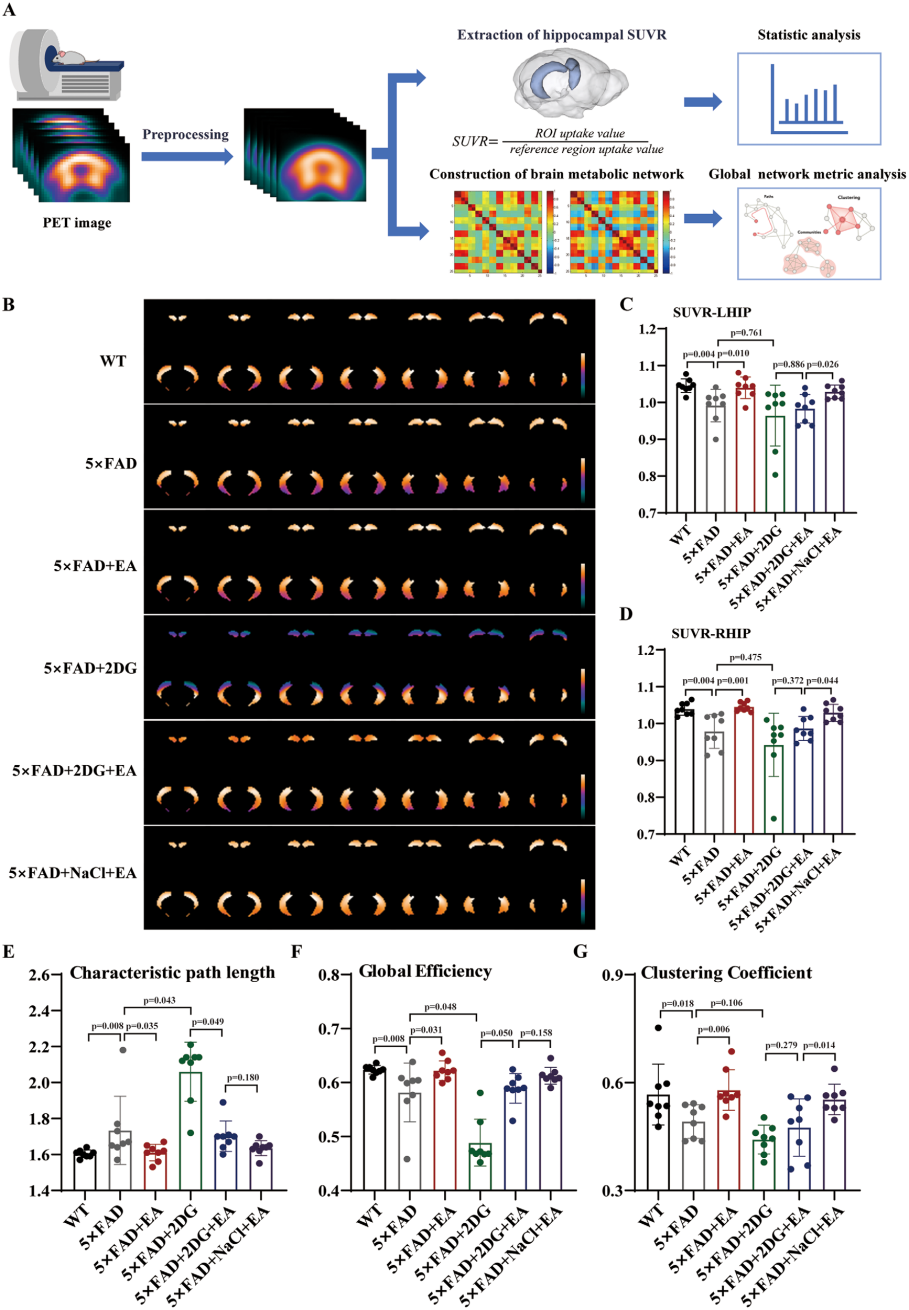
EA improved the brain glucose metabolic network in the 5×FAD mice. (A) PET data analysis diagram. (B) Representative images of the SUVR value in the bilateral hippocampus. (C,D) Quantification of SUVR in the hippocampus (*n* = 8 mice/group). (E–G) The characteristic path length (E), global efficiency (F), and clustering coefficient (G) of the brain glucose metabolic network (*n* = 8 mice/group). All data were shown as mean ± SEM. *P* values were indicated in the figure. Statistical analysis was assessed with the Kruskal‐Wallis H test (C‐F), one‐way ANOVA with LSD comparisons (G).

FDG‐PET imaging is used to quantify brain glucose metabolism through radiotracer accumulation patterns [22, 23]. It showed that EA significantly up‐regulated the SUVR signal of the hippocampus in 5×FAD mice (Figure 2A–D). Following the observed cognitive enhancements and the regulation of hippocampal glucose levels after EA intervention, we further investigated the brain glucose metabolic network. This network represents the functional synergy between various brain regions based on the metabolic activities. Compared with the AD group, EA significantly decreased the characteristic path length of the whole‐brain network (Figure 2E), increased the global efficiency (Figure 2F), and clustering coefficient (Figure 2G). These data demonstrate that EA alleviates the information integration and transmission efficiency of the brain network in 5×FAD mice. Notably, the inhibition of hippocampal glucose metabolism by 2DG could abolish the topological improvements, suggesting that EA‐mediated network remodeling is dependent on the glucose metabolic enhancement.

### 2.3 EA Improved the Hippocampal Glucose Metabolism in AD Mice

**FIGURE 3:**
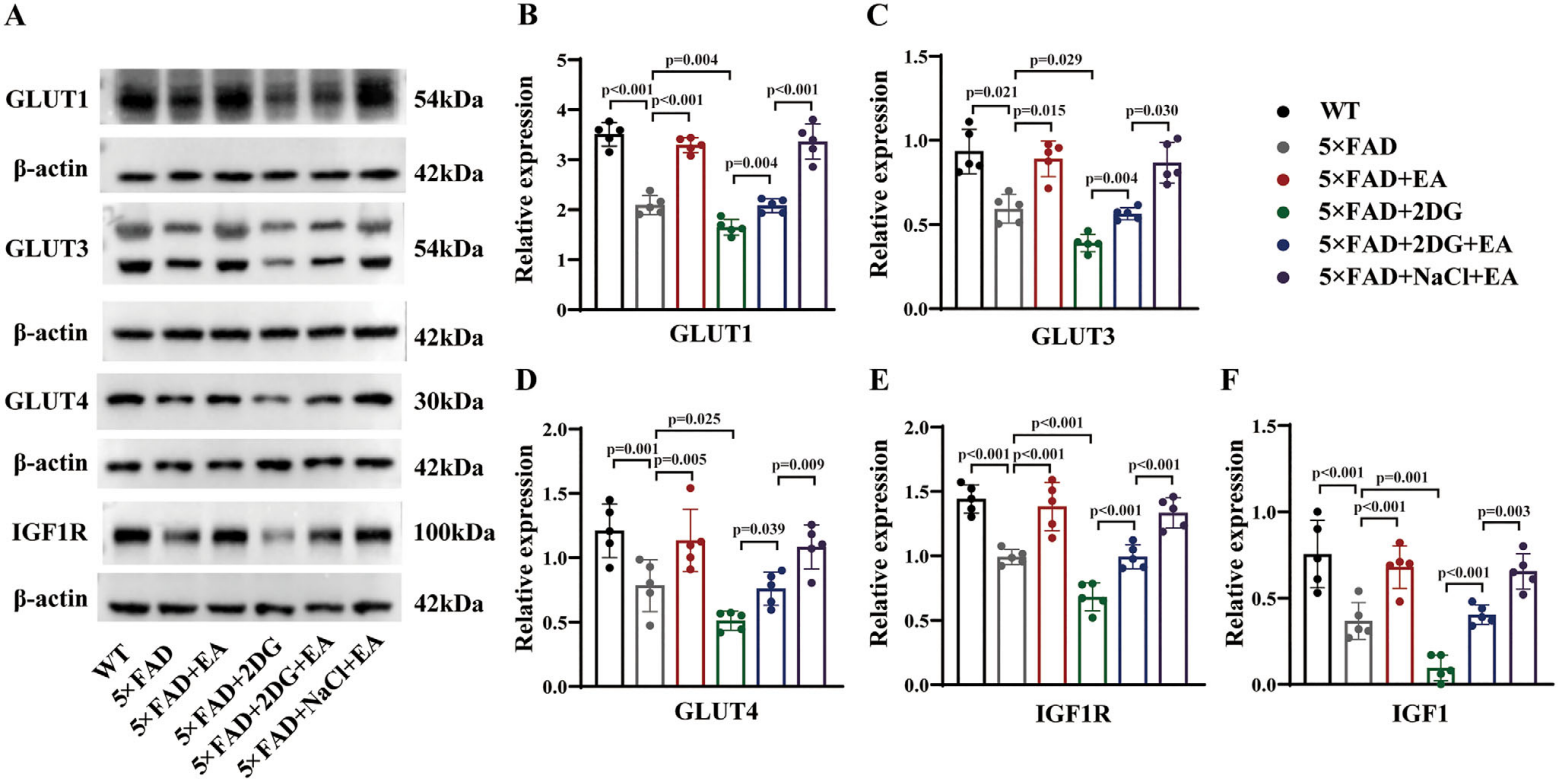
EA enhanced the hippocampal glucose metabolism in 5×FAD mice. (A) Representative western blotting patterns of GLUT1, GLUT3, GLUT4, IGF1R, and β‐actin in the hippocampus. (B–E) Quantification of GLUT1, GLUT3, GLUT4, and IGF1R in the hippocampus (*n* = 5 mice/group). (F) Relative amounts of IGF1 quantified by ELISA (*n* = 5 mice/group). All data were shown as mean ± SEM. *P* values were indicated in the figure. Statistical analysis was assessed with one‐way ANOVA followed by LSD comparisons test (B, D, E, and F) and Dunnett's T3 post‐hoc test (C). GLUT1: Glucose transporter 1; GLUT3: Glucose transporter 3; GLUT4: Glucose transporter 4; IGF1R: Insulin‐Like Growth Factor 1 Receptor; IGF1: Insulin‐Like Growth Factor 1.

Abnormal hippocampal glucose metabolism in AD is characterized by diminished glucose uptake. Neurons in the brain cannot directly synthesize or store glucose. It needs to continuously take in glucose through glucose transporters to meet the energy demands for neural activity. EA significantly increased the expression of GLUT1, GLUT3, and G LUT4 in the hippocampus (Figure 3A–D). GLUT3 is a key transporter protein specifically expressed by neurons for glucose uptake. IGF1/IGF1R is an upstream signal of GLUT3. Correspondingly, EA increased the expression of IGF1R (Figure 3E) and IGF1 (Figure 3F) of the hippocampus in 5×FAD mice. Taken together, EA might improve glucose metabolism of the hippocampus in AD by enhancing the expression of GLUT3 and IGF1/IGF1R.

### 2.4 EA Improved the Learning and Memory of AD Mice via Regulating Hippocampal IGF1R

**FIGURE 4:**
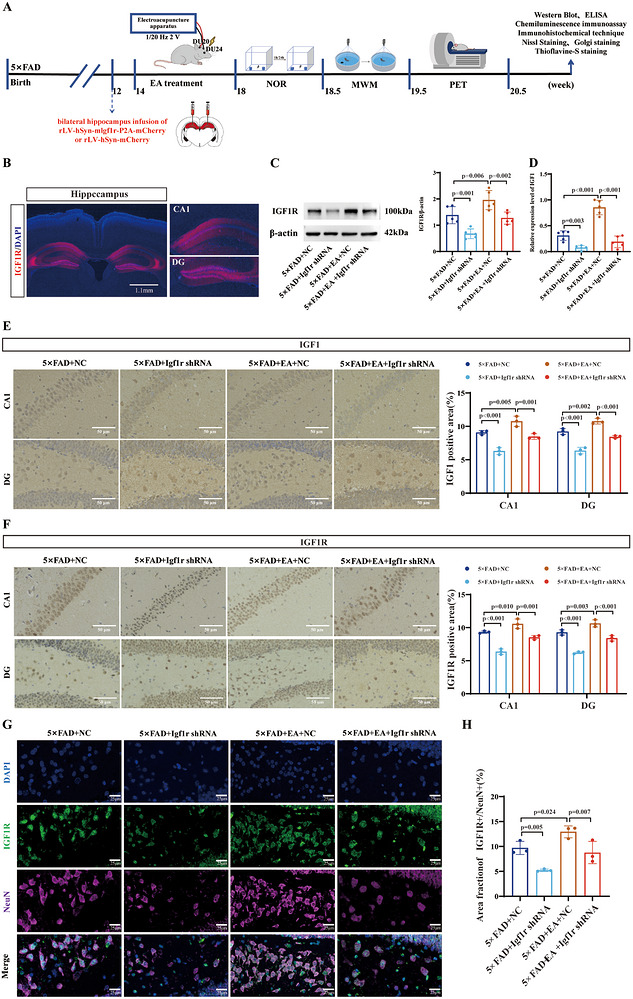
The effects of EA on IGF1 and IGF1R expression. (A) Experimental procedures. (B) Representative images of rLV‐Igf1r shRNA in the hippocampus (scale bar, 1.1 mm). (C) Representative immunoblots of IGF1R (left) and quantification of IGF1R in the hippocampus (right) (*n* = 5 mice/group). (D) Relative amounts of IGF1 quantified by ELISA (*n* = 5 mice/group). (E,F) Representative immunohistochemistry images of the IGF1 (E left) and IGF1R (F left) (scale bars, 50 µm), and quantification of the positive areas for IGF1 (E right) and IGF1R (F right) in the hippocampus (*n* = 3 mice/group, each n represents the average of three technical replicates). (G) Representative images of the IGF1R (green) in neurons (rose red), DAPI (blue), and their colocalization in the hippocampus (scale bars, 25 µm). (H) Quantification of IGF1R‐positive neurons in the hippocampus (*n* = 3 mice/group, each n represents the average of three technical replicates). All data were shown as mean ± SEM. *P* values were indicated in the figure. Statistical analysis was assessed with one‐way ANOVA followed by the LSD comparisons test (C‐F, H). IGF1R: Insulin‐Like Growth Factor 1 Receptor; IGF1: Insulin‐Like Growth Factor 1; NeuN: Neuronal Nuclear Antigen.

To evaluate whether inhibiting IGF1R could counteract the protective effect of EA on cognitive function in 5×FAD mice, we injected rLV‐hSyn‐mlgf1r‐P2A‐mCherry or the rLV‐hSyn‐mCherry into the hippocampus in 5×FAD mice (Figure 4A). The expression of IGF1R was confirmed by fluorescence imaging, western blot, and immunohistochemistry in the hippocampus (Figure 4B,C,F–H). Besides, EA significantly increased the expression levels of IGF1 in the hippocampus (Figure 4D,E). However, after specifically inhibiting the expression of IGF1R, the upregulation effect was significantly weakened.

**FIGURE 5:**
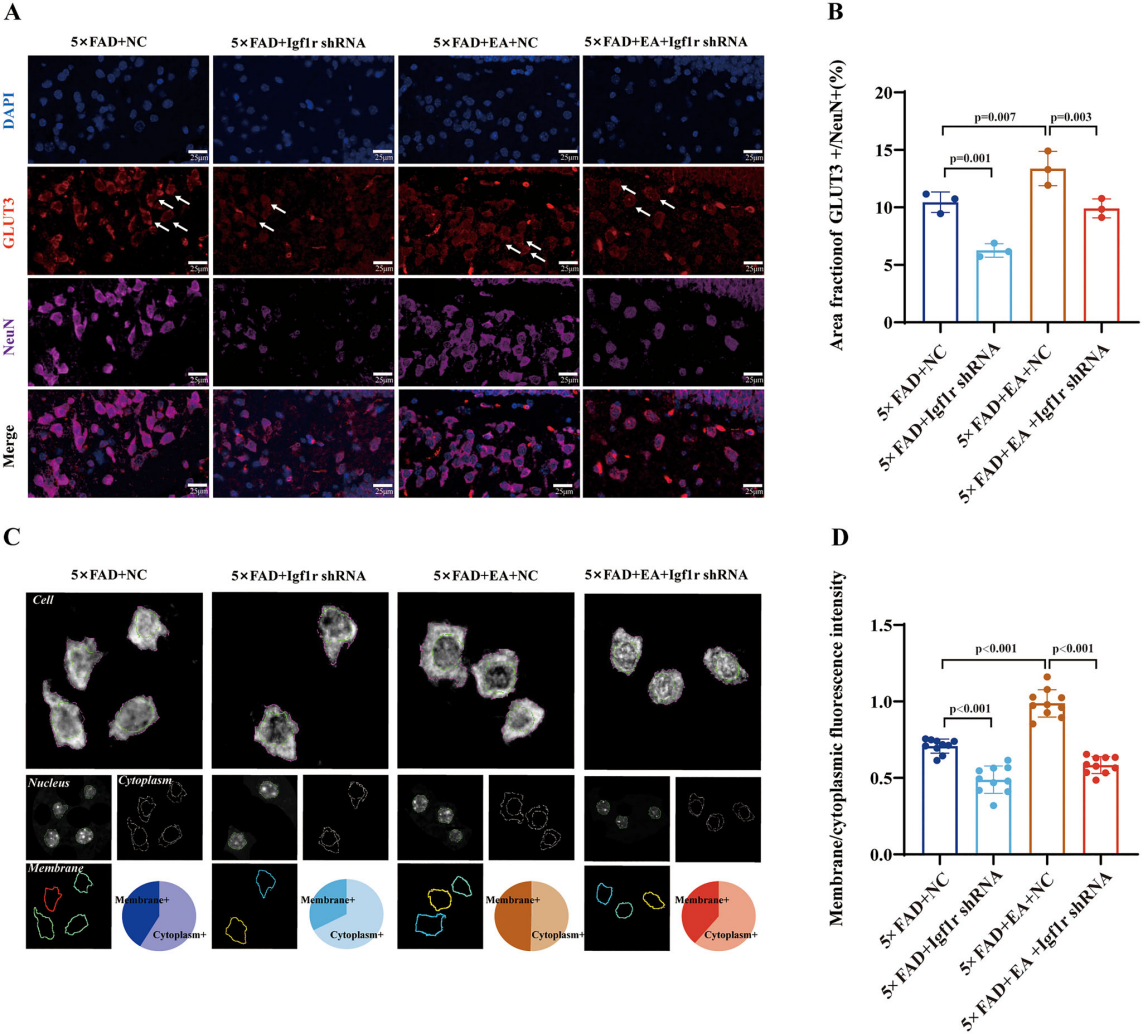
EA promoted GLUT3 membrane translocation mediated by IGF1R in 5×FAD mice. (A) Representative images show the GLUT3 (green) in neurons (rose red), DAPI (blue), and their colocalization in the hippocampus (scale bars, 25 µm). (B) Quantification of GLUT3‐positive neurons in the hippocampus (*n* = 3 mice/group, each n represents the average of three technical replicates). (C, D) Quantification of the membrane‐to‐cytosol ratio of GLUT3 fluorescence intensity in hippocampal neurons (*n* = 10 cells/group). All data were shown as mean ± SEM. *P* values were indicated in the figure. Statistical analysis was assessed with one‐way ANOVA followed by the LSD comparisons test (B and D). GLUT3: Glucose transporter 3; NeuN: Neuronal Nuclear Antigen.

**FIGURE 6:**
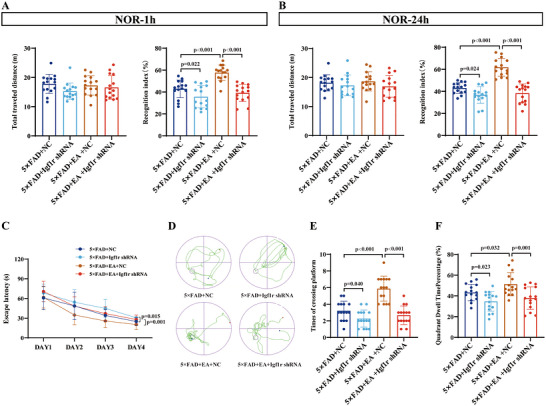
IGF1R deficiency diminished the effects of EA on cognitive function in 5×FAD mice. (A,B) Motor ability was comparable across all groups (A, left; B, left), and recognition index was assessed by the NOR test at 1 h (A, right) and 24 h (B, right) after rLV‐Igf1r shRNA infection in the hippocampus (*n* = 15 mice/group). (C) Time spent before reaching the hidden platform in the MWM. (D) Representative swimming traces of 4 groups during the probe trial. (E) Number of entries into the platform during the probe trial. (F) Time spent in the target quadrant during the probe trial (*n* = 15 mice/group). All data were shown as mean ± SEM. *P* values were indicated in the figure. Statistical analysis was assessed with one‐way ANOVA followed by LSD comparisons test(A–F).

In addition, immunofluorescence analysis showed that EA could specifically enhance the membrane localization level of GLUT3 (Figure 5A–D) in NeuN‐positive neurons of the hippocampus. Inhibition of IGF1R expression significantly attenuated the EA‐induced membrane localization of GLUT3. Besides, compared with the mice injected with Igf1r shRNA virus, the mice injected with the control virus exhibited better cognitive behavioral performance after EA (Figure 6A–F). In the NOR experiment, mice in the control virus with EA intervention group exhibited a significantly higher discrimination index (Figure 6A,B). Similarly, in the MWM test, mice in the control virus with EA intervention group showed a markedly shortened escape latency (Figure 6C). Furthermore, probe trial results indicated that IGF1R inhibition abolished the memory‐enhancing effects of EA, as evidenced by fewer platform crossings and reduced time spent in the target quadrant (Figures 6D–F). Thus, EA significantly improved the cognitive function in 5×FAD mice, and this effect might depend on the IGF1R signaling pathway.

**FIGURE 7:**
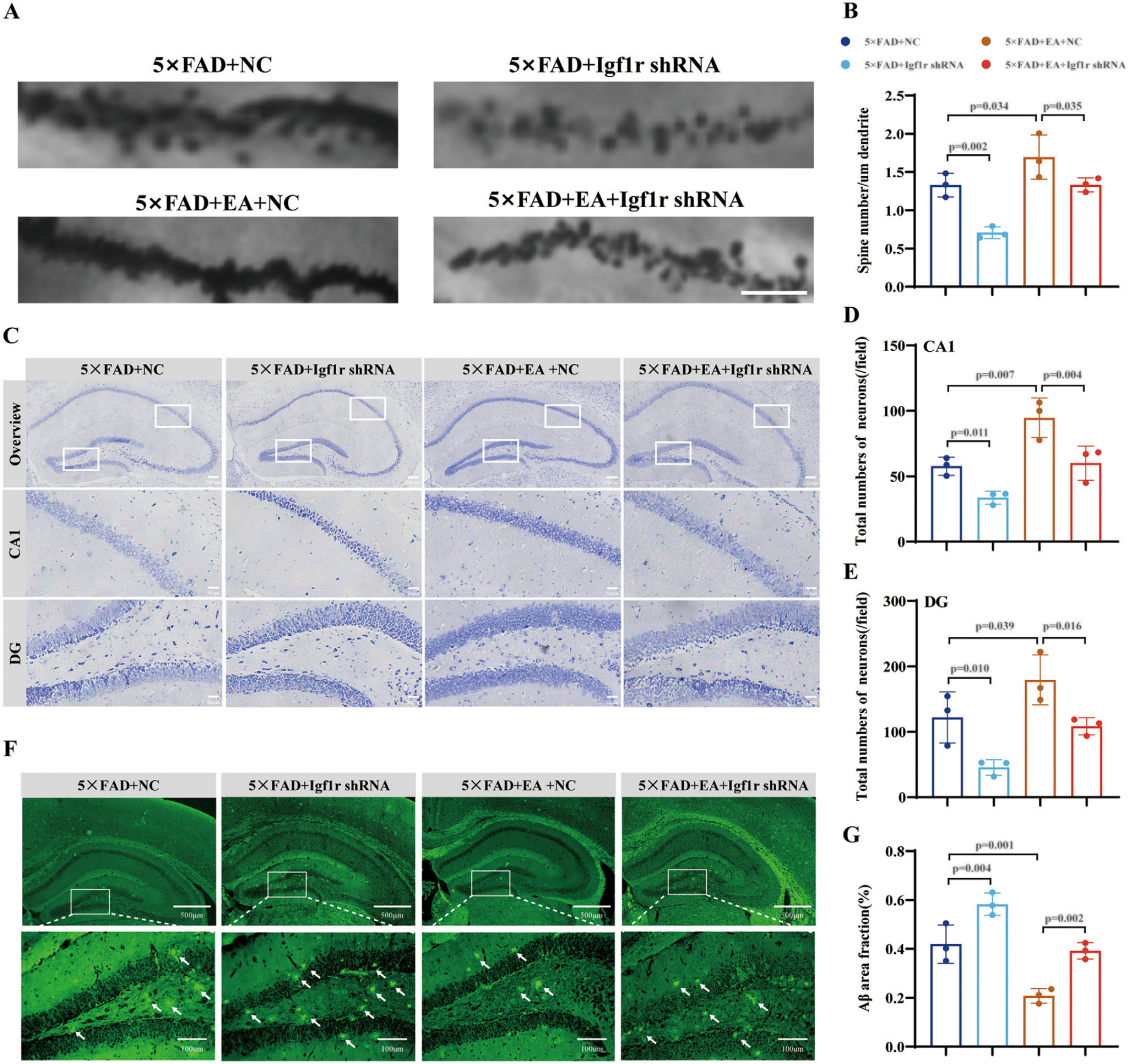
IGF1R knockdown attenuates the neuroprotective effects of EA in 5×FAD mice. (A) Representative images of Golgi staining in the hippocampus (scale bars, 5 µm). (B) Quantification of hippocampal dendritic spines (*n* = 3 mice/group, each n represents the average of three technical replicates). (C) Representative images of Nissl staining in the hippocampus (scale bars, 50 µm). (D,E) Quantification of the intact neurons in the area framed within a white bordered rectangle in the CA1 (D) and DG (E) region of the hippocampus (*n* =  3 mice/group, each n represents the average of three technical replicates). (F) Representative images of Aβ (scale bars, 500 µm, 100 µm). (G) Quantification of Aβ area in the hippocampus (*n* = 3 mice/group, each n represents the average of three technical replicates). All data were shown as mean ± SEM. *P* values were indicated in the figure. Statistical analysis was assessed with one‐way ANOVA followed by the LSD comparisons test (B, D, and G).

Using multimodal histological analysis, our study demonstrated that EA mediates its neuroprotective effects via activation of the IGF1R. Golgi staining showed that IGF1R inhibition reduced the increase in dendritic spinous density in the hippocampus by EA (Figure 7A,B). Nissl staining confirmed that IGF1R was crucial for EA to maintain neuronal density and structural integrity (Figure 7C–E). Thioflavin S staining indicated that IGF1R inhibition would reduce the clearance of Aβ deposits in the hippocampus by EA (Figure 7F,G). Overall, these findings reveal that IGF1R is important in mediating the effects of EA on improving the learning and memory, regulating synaptic plasticity, and alleviating pathological alterations in 5×FAD mice.

### 2.5 EA Improved the Hippocampal IGF1/IGF1R Signaling Pathway and Enhanced Glucose Metabolic Network in AD Mice

**FIGURE 8:**
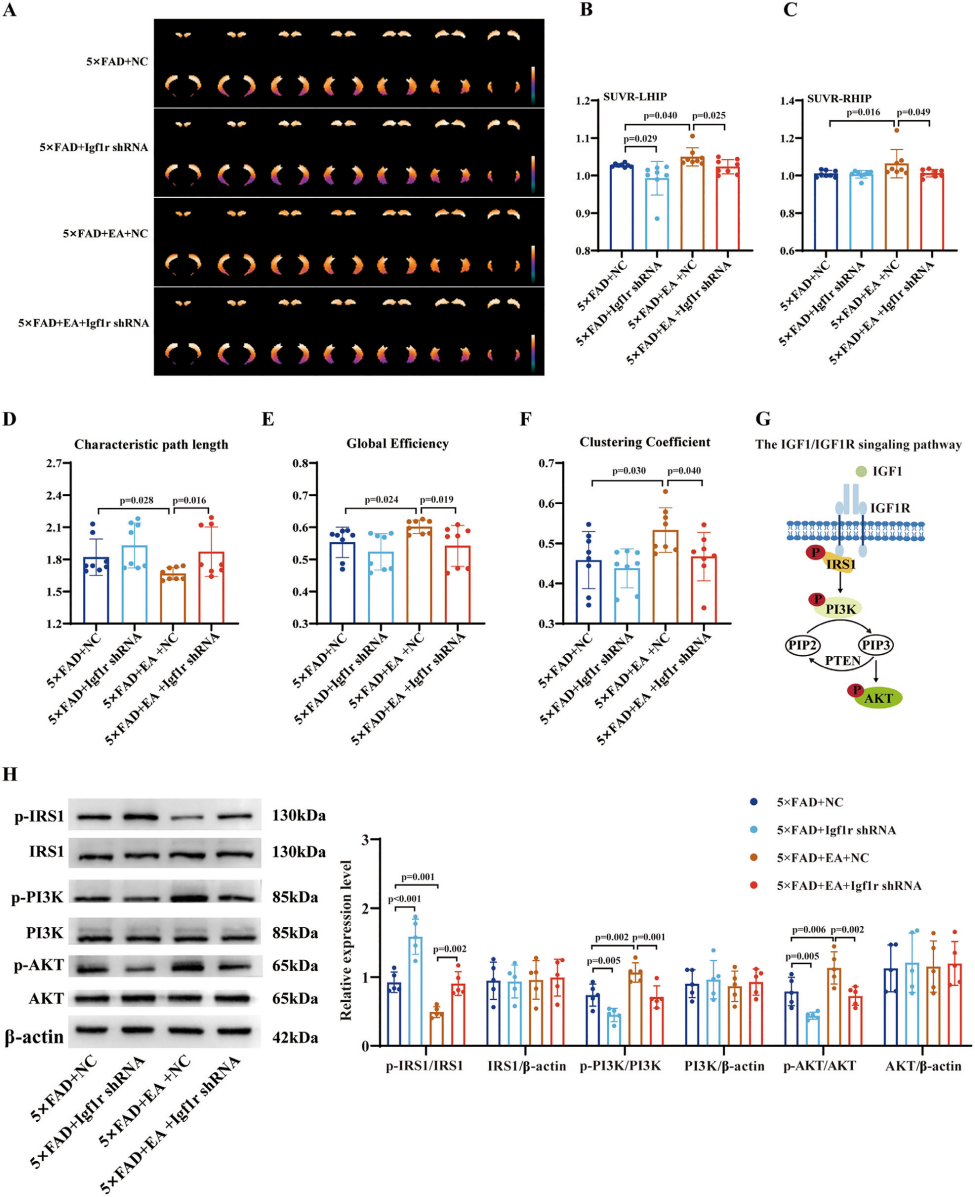
EA improved hippocampal IGF1/IGF1R signaling pathway and enhanced glucose metabolic network in 5×FAD mice. (A) Representative images of the SUVR value in the bilateral hippocampus. (B,C) Quantitation of SUVR in the bilateral hippocampus (*n* = 8 mice/group). (D–F) The characteristic path length (D), global efficiency (E), and clustering coefficient (F) of the brain glucose metabolic network (*n* = 8 mice/group). (G) Schematic diagram of the IGF1/IGF1R signaling pathway. (H) Representative western blotting patterns of p‐IRS1, IRS1, p‐PI3K, PI3K, p‐AKT, AKT, and β‐actin in the hippocampus (left), and quantification of the relative levels (right) (*n* = 5 mice/group). All data were shown as mean ± SEM. *P* values were indicated in the figure. Statistical analysis was assessed with the Kruskal‐Wallis H test (B‐F), one‐way ANOVA with LSD comparisons (H). IGF1R: Insulin‐Like Growth Factor 1 Receptor; IGF1: Insulin‐Like Growth Factor 1; p‐IRS1: Phosphorylated insulin receptor‐1‐substrate; IRS1: Insulin receptor substrate 1; p‐PI3K: Phosphorylated phosphatidylinositol‐3‐kinase; PI3K: Phosphatidylinositol 3‐kinase; p‐AKT: Phosphorylated protein kinase B; AKT: Protein kinase B.

FDG‐PET imaging revealed that hippocampal IGF1R knockdown significantly attenuated the stimulatory effects of EA on glucose metabolism, accompanied by a reduction in bilateral hippocampal SUVR (Figure 8A–C). Further analysis of brain network topology revealed that the specific inhibition of IGF1R expression significantly attenuated the beneficial effects of EA (Figure 8D–F). Therefore, IGF1R is required for EA to optimize the integration and segregation properties of the brain network.

To explore the molecular mechanism by which IGF1R regulates GLUT3, we examined the expression of proteins related to the IGF1R signaling pathway in the hippocampus. We found that EA could specifically reduce the phosphorylation level at the Ser307 site of the IRS1 protein, and significantly enhance the phosphorylation modification of PI3K Tyr458 and AKT Ser473. When the expression of IGF1R was specifically inhibited, the effects of EA on p‐IRS1, p‐PI3K, and p‐AKT were decreased (Figure 8G,H).

### 2.6 EA Improved Energy Supply in the Hippocampus of AD Mice

**FIGURE 9:**
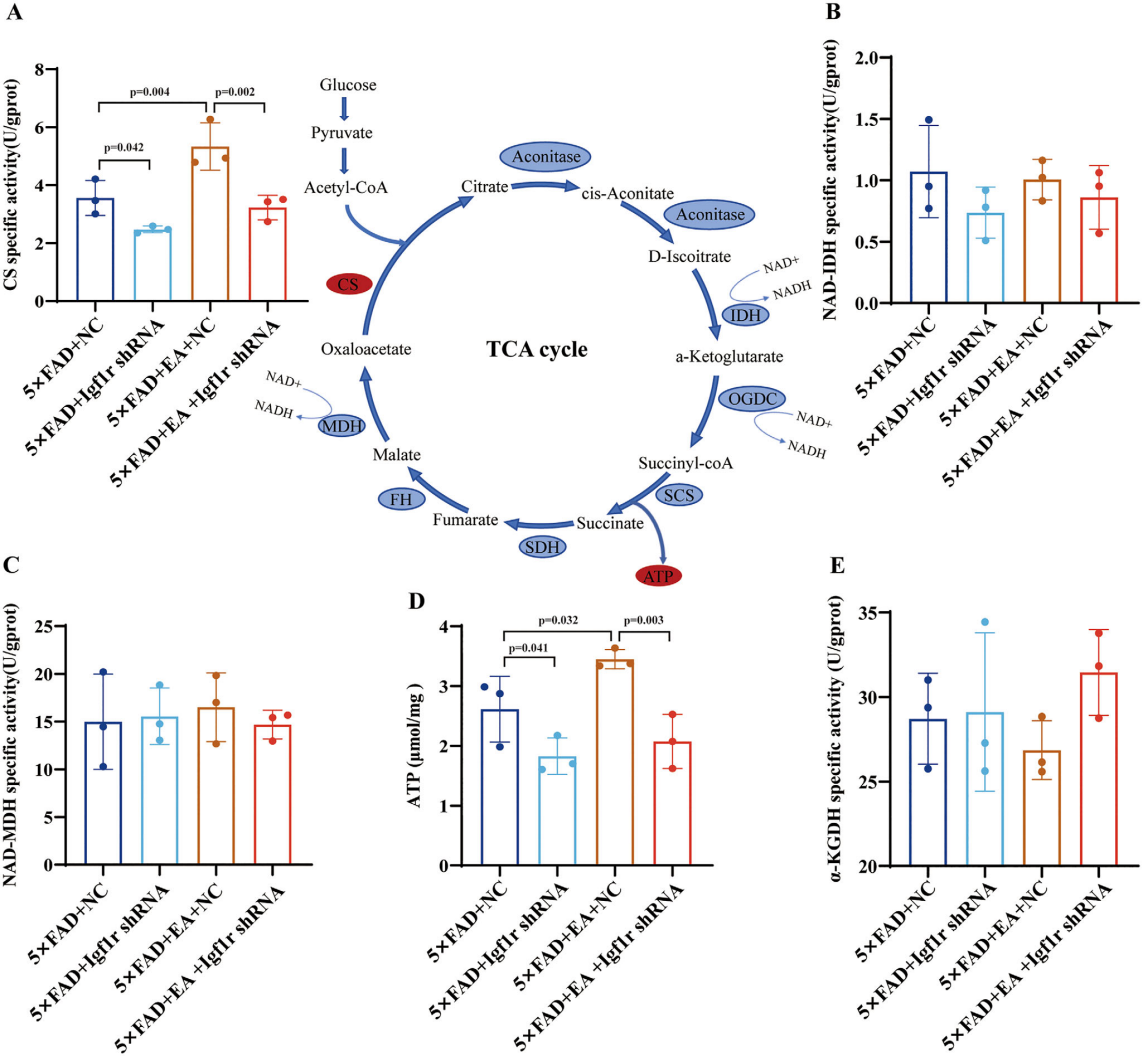
EA modulated the enzyme activities related to the tricarboxylic acid cycle and ATP content in the hippocampus. (A–E) Quantification of enzyme activities and ATP content, including CS (A), NAD‐IDH (B), NAD‐MDH (C), ATP content (D), and α‐KGDH (E) in the hippocampus (n  =  3 mice/group). All data were shown as mean ± SEM. *P* values were indicated in the figure. Statistical analysis was assessed with one‐way ANOVA followed by LSD comparisons test(A–E). CS: citrate synthase; NAD‐MDH: NAD‐dependent malate dehydrogenase; NAD‐IDH: NAD‐dependent isocitrate dehydrogenase; α‐KGDH: α‐ketoglutarate dehydrogenase; ATP: Adenosine 5'‐triphosphate.

The tricarboxylic acid cycle plays a crucial role in glucose metabolism. As the first rate‐limiting enzyme in the TCA cycle, the activity of citrate synthase directly affects the ATP generation efficiency. Quantitative analysis showed that EA specifically enhanced the citrate synthase activity and improved the ATP production. IGF1R plays a critical role in EA improving these indicators, while it showed no significant effect on isocitrate dehydrogenase, α‐ketoglutarate dehydrogenase, or malate dehydrogenase activities (Figure 9A–E).

## 3 Discussion

We found that EA improves the learning and memory in 5×FAD mice by regulating glucose metabolism in the hippocampus. Glucose metabolism disorder in the hippocampus is the core pathological feature of cognitive dysfunction in AD, which is instrumental in neural information processing and memory consolidation [24]. It is worth noting that both AD patients and animal models exhibit significant abnormalities in hippocampal glucose metabolism [25, 26, 27]. Alleviating hippocampal glucose metabolism could reduce cognitive impairment in AD [8].

During AD progression, the brain glucose metabolic network undergoes obvious degenerative alterations. These changes are characterized by reduced metabolic network and disrupted balance of local network properties [28, 29]. Our study found that EA could significantly restore the global efficiency and clustering coefficient, shorten the characteristic path length in 5×FAD mice. This high clustering coefficient and short characteristic path lengths reflect enhanced capacity for both specialized local processing and efficient parallel information integration across the whole brain [30, 31]. The characteristic path length quantifies the average minimum number of edges between any two nodes. The shorter path length means higher communication efficiency across brain regions and better cognitive function. Global efficiency characterizes the overall capacity of a network for parallel information transmission. The clustering coefficient reflects the level of local interconnection within the network [32]. In AD, the decline in global efficiency correlates significantly with the severity of cognitive impairment [33], and a reduced clustering coefficient serves as a sensitive predictor of AD onset risk [34]. It is noteworthy that inhibition of hippocampal glucose metabolism weakens the ability of EA to improve whole‐brain topological properties. This indicates that EA regulates cognitive function in AD, and this regulatory effect is largely dependent on hippocampal energy homeostasis.

Hippocampal glucose metabolism is severely impaired in AD. This dysfunction involves reduced glucose uptake and disrupted oxidative decomposition. Our study found that EA could mediated GLUT3 membrane translocation, increased SUVR values in the hippocampus, and improved cognitive function in 5×FAD mice. GLUT3, as the primary neuronal glucose transporter, exhibits high affinity and ensures a stable energy supply even under low‐glucose conditions [35, 36]. GLUT3 predominantly resides in the cytoplasm in a functionally inactive state. In response to neural activity, GLUT3 would insert into the cell membrane. It then facilitates efficient glucose uptake into cells, which provides energy for synaptic transmission [37]. Our study also found that this process depends on the integrity of the IGF1/IGF1R signaling pathway.

The IGF1/IGF1R signaling pathway serves as a central regulatory axis for brain energy homeostasis and cognitive integrity [38]. IGF1 is a biomarker of cognition and the IGF1 signaling pathway represents a possible therapeutic target to prevent or slow age‐related cognitive decline [39]. Our study found that EA could activate the IGF1/IGF1R signaling pathway of the hippocampus, restore glucose metabolism, and ameliorate cognitive deficits in 5×FAD mice. Although IGF1 is primarily secreted by the liver, the brain regions, including hippocampus, cerebellum, and subventricular zone, can also generate IGF1 under the stimulation of growth hormone [40]. Notably, the ratio of hippocampal active/inactive IGF1 in AD patients is reduced by 60% [41]. We found that IGF1R expression in the hippocampus was decreased, which may be attributable to the reduction in the number of IGF1R‐expressing neurons in 5×FAD mice. The IGF1/IGF1R/AKT signaling pathway plays roles beyond metabolic regulation. It also maintains neuronal homeostasis by controlling autophagy. When the AKT activation cascade is disrupted, it would lead to synaptic loss and cognitive decline in AD [42]. Despite central and peripheral administration of IGF1 showed significant neuroprotective effects, peripheral administration could reduce the cognitive impairment in the chronic phase of cerebral ischaemia [43].

Our study also found that EA reduced the phosphorylation level of IRS1, enhanced the phosphorylation levels of PI3K and AKT. This series of molecular events promoted the transport of GLUT3 to the neuronal plasma membrane. It improved glucose metabolism and energy supply in neurons. In addition, abnormal serine phosphorylation of IRS1 in the brain is negatively associated with cognitive function, and positively associated with Aβ plaques and neurofibrillary tangles [44, 45]. Glucose metabolism in the hippocampus was affected by PI3K inhibitors in AD mice [46]. In addition, it has been reported that the expression of α‐ketoglutarate dehydrogenase and malate dehydrogenase, the key enzymes of the tricarboxylic acid cycle, was decreased in AD mice [47, 48]. ApoE4‐carrying mice exhibited age‐dependent deterioration in hippocampal transcription and metabolism, characterized by diminished citrate synthase activity and reduced ATP levels [49]. Research has found that high‐frequency rTMS could improve glucose metabolism in 3×Tg‐AD mice and reduce Aβ deposition in the hippocampus by modulating the PI3K/AKT signaling pathway [50]. Furthermore, physical exercise could upregulate major biomarkers of neuroplasticity, such as IGF1 and BDNF. This process activates the downstream PI3K/Akt signaling pathway to support cognitive function [51, 52]. Therefore, the IGF1/IGF1R signaling pathway may be an effective approach to improve learning and memory in AD.

Our research has several limitations. First, although our study found that EA could promote the activity of citrate synthase and improve energy metabolism, it did not examine the molecular mechanism of energy metabolism in mitochondria. Second, our results suggest that EA effectively modulates hippocampal glucose metabolism and the topology of the brain metabolic network; the mechanisms underlying its coordination of metabolic synchrony and functional connectivity across distinct brain regions remain to be fully unelucidated. Besides, it is needed to investigate the source of IGF1 modulated by EA. In addition, further study should apply a rescue experiment to validate the causal sufficiency between the IGF1/IGF1R signaling pathway and cognition in AD.

In summary, our results revealed that EA may promote GLUT3‐mediated glucose uptake and energy metabolism by activating the IGF1/IGF1R signaling pathway in the hippocampus, enhance brain network information transmission and integration, and improve learning and memory in 5×FAD mice. This discovery highlights the therapeutic value of targeting the IGF1/IGF1R signaling system in treating cognitive impairment and provides a new theoretical basis for the development of acupuncture‐based intervention strategies for neurodegenerative diseases.

## 4 Methods

### 4.1 Animals

The male 5×FAD mice (Jiangsu Wukong Biotechnology Co., LTD, China) display overexpression of APP and PSEN1 [53], and wild‐type (WT) C57BL/6 female mice were bred; the genotyping of the offspring was detected using polymerase chain reaction (PCR) analysis on tail DNA samples. The mice were housed 3–5 per cage and maintained under the standard environmental conditions, with a 12‐h alternating light/dark schedule, room temperature maintained at 21±1°C, and plenty of food and water. The study received approval from the Ethical Committee on Animal Experimentation at Fujian University of Traditional Chinese Medicine (FJTCMIACUC2021001).

### 4.2 Experiment Design

Mice were randomly assigned to the experimental groups using a computer‐generated random number table to ensure unbiased allocation. To maintain objectivity, a blind protocol was implemented throughout the study. The researchers administering the EA intervention were not involved in subsequent behavioral testing and molecular assays. Furthermore, all data collection and analysis were performed by investigators who were blinded to the experimental grouping.

This study was divided into two parts. First (Figure 1A), to assess the improvement of the learning and memory, hippocampal function, and glucose metabolism in AD mice, the 3‐month‐old 5×FAD mice were randomly divided into five groups (*n* = 10/group): the 5×FAD group, 5×FAD+EA group, 5×FAD+2DG group, 5×FAD+2DG+EA group, and 5×FAD+NaCl+EA group, additionally, age‐matched WT mice were used as the controls.

Second (Figure 4A), to reveal the effect of EA mediated hippocampal glucose metabolism regulating IGF1/IGF1R signaling pathway in AD mice, the 3‐month‐old 5×FAD mice were divided into four groups (*n* = 15/group): the 5×FAD+control virus group (5×FAD+NC), 5×FAD+Igf1r shRNA virus group (5×FAD+Igf1r shRNA), 5×FAD+EA+control virus group (5×FAD+EA+NC), and 5×FAD+EA+Igf1r shRNA virus group (5×FAD+EA+Igf1r shRNA).

### 4.3 Virus Injection

First, the glucose metabolism inhibitor 2DG (Beyotime Biotech, Shanghai, China) [54] was bilaterally microinjected into the hippocampus. Mice were anesthetized with 5% isoflurane and placed in a prone position on a stereotaxic instrument. An oxygen mask was put into the nose tip to maintain anesthesia (1.5% isoflurane) throughout the surgery. The head was fixed with a dental rod and an ear rod. The customized guide cannula was positioned in the specified brain regions, utilizing coordinates derived from the Paxinos and Franklin stereotaxic atlas [55]: hippocampus, AP:‐2.1 mm, ML:±1.4 mm, DV:‐2 mm. Burr holes were drilled over the target brain regions. A customized cylindrical plastic sleeve made of polyvinyl chloride (RWD Life Science; C: 1.7, G1: 0.5, G2: 0.5) was then secured to the skull using acrylic dental cement. The 2DG dissolved in 0.9% NaCl was injected into the bilateral hippocampus of the 5×FAD+2DG group and the 5×FAD+2DG+EA group. The 0.9% NaCl was injected into the same location of 5×FAD+NaCl+EA group. The solution was infused at a rate of 30–40 nL/min over 10 min, yielding a total volume of 300–400 nL per infusion. The syringe was then raised 0.02 mm and left in place for an additional 10 min for its diffusion before being slowly withdrawn. Prior to each EA session, either the inhibitor or normal saline was injected.

Second, all viruses were obtained from Brain Case (ShenZhen, China), and we refer to the literature to establish a protocol for virus sequence interference and injection. The rLV‐hSyn‐mlgf1r‐P2A‐mCherry virus was injected into the bilateral hippocampus of mice in the 5×FAD+Igf1r shRNA and 5×FAD+EA+Igf1r shRNA groups. The rLV‐hSyn‐mCherry virus was injected into the same region in the 5×FAD+NC and 5×FAD+EA+NC groups. The hippocampal injection coordinates relative to Bregma were as follows: AP:‐2.1 mm, ML:±1.4 mm, DV:‐2 mm, and AP:‐2.9 mm, ML:±3 mm, DV:‐3.8 mm [56]. The virus was infused at a rate of 50 nL/min over 10 min, yielding a total volume of 500 nL [57]. The needle was then raised 0.02 mm and left in place for 10 min for viral spread before being slowly withdrawn. Viral expression was confirmed by fluorescence microscopy 15 day.

### 4.4 EA Intervention

Following a previously established protocol [58], needles were inserted into the Baihui (DU20) and Shenting (DU24) acupoints. Electrical stimulation was delivered at 2/20 Hz and 1 mA for 30 min/day, 5 days/week, over 4 weeks in the 5×FAD+EA, 5×FAD+2DG+EA, 5×FAD+NaCl+EA, 5×FAD+EA+NC, and 5×FAD+EA+Igf1r shRNA groups. Mice in the WT, 5×FAD, 5×FAD+2DG, 5×FAD+NC, and 5×FAD+Igf1r shRNA groups were restrained under identical conditions without EA intervention.

### 4.5 Behavioral Analysis

#### 4.5.1 Novel Object Recognition (NOR) Test

The NOR test (Figure 1C,D) was used to assess the learning and memory function [59]. On the first day, all mice were placed in the open‐field box for 10 min prior to the task to acclimate to the environment. In the training session, mice were allowed to explore two identical objects for 10 min. At 1 h and 24 h after training, one object was replaced with a novel object, and mice were allowed to explore the two objects for 5 min. The recognition index was calculated as the time spent exploring the novel object divided by the total exploration time.

#### 4.5.2 Morris Water Maze (MWM)

The MWM (Figure 1G) was conducted over 6 days to assess hippocampus‐dependent spatial learning and memory [60]. On the first day, all mice were placed in the pool filled with water at (22 ± 2)°C for 3 min to acclimate to the environment. A platform (6 cm in diameter) was submerged 1 cm below the opaque water surface in the third quadrant of the pool. During the acquisition phase, mice were trained to locate the hidden platform over four trials per day for 4 consecutive days. In the probe trial, the platform was removed, and mice were allowed to swim freely for 90 s. The number of platform crossings and the percentage of time spent in the target quadrant were analyzed.

### 4.6 PET Imaging and Analysis

Eight mice per group were randomly selected for PET scanning, which was performed on a Siemens PET‐CT scanner at the Small Animal PET Center, Department of Nuclear Medicine, Beijing Anzhen Hospital. The radiotracer 18F‐FDG was used for all scans. Mice were fasted for 24 h prior to scanning. 18F‐FDG was administered via tail vein injection, after which mice were allowed to move freely in their cages for 40 min to allow for tracer uptake and distribution. Mice were then anesthetized with 1.5%–2% isoflurane, placed in a prone position on the scanning bed, and their heads were secured using ear and tooth bars. CT images were acquired first, followed by PET image acquisition over 10 min. The image matrix size was 256 × 256 × 159, with a resolution of 0.4 × 0.4 mm^2^ and a slice thickness of 0.8 mm. PET images were reconstituted using a 3D ordered‐subset expectation maximization (3D‐OSEM) algorithm (21 subsets, 2 iterations) with CT attenuation correction and isotope decay correction.

All images were preprocessed using SPM12 (London, United Kingdom) (Figure 2A). Preprocessing steps included skull stripping, origin correction, spatial normalization to the mouse brain template [61], and smoothed with a 0.6 mm FWHM kernel. SUVR values for each brain region were extracted following normalization to whole‐brain white matter [62, 63, 64, 65]. To generate the brain metabolic network, 25 regions of interest were selected as nodes [61]. Then, a 25 × 25 glucose metabolic connectivity matrix was constructed [66]. Finally, the network indicators, including characteristic path length, global efficiency, and clustering coefficient were estimated using GRETNA (https://www.nitrc.org/projects/gretna/).

### 4.7 Western Blot

Hippocampal tissues were homogenized in radioimmunoprecipitation assay (RIPA) buffer supplemented with a phosphatase and protease inhibitor cocktail. Total protein concentration was determined by BCA assay and adjusted to 5 µg/µl. Proteins were separated by 10% SDS‐PAGE, transferred to polyvinylidene fluoride (PVDF) membranes, and blocked with 5–8% non‐fat milk for 3 h. Membranes were then incubated overnight at 4°C with the following primary antibodies: β‐actin (Proteintech, 20536‐1‐AP, 1:1000), GLUT1 (Abcam, ab115730, 1:1000), GLUT3 (Abcam, ab191071, 1:1000), GLUT4 (Abcam, ab33780, 1:1000), IGF1R (Abcam, ab182408, 1:1000), IRS (Cell Signaling Technology, 2382S, 1:500), p‐IRS (Cell Signaling Technology, 2381S, 1:500), AKT (Cell Signaling Technology, 4691S, 1:1000), p‐AKT (Cell Signaling Technology, 4060S, 1:1000), PI3K (Cell Signaling Technology, 4292S, 1:1000), and p‐PI3K (Cell Signaling Technology, 17366S, 1:1000). After washing, membranes were incubated with a goat anti‐rabbit secondary antibody (Proteintech, SA00001‐2, 1:5000) at room temperature for 1 h. Protein bands were visualized by enhanced chemiluminescence (ECL) and quantified using Image Lab software. Relative protein expression was normalized to β‐actin or the corresponding total protein.

### 4.8 Enzyme‐Linked Immunosorbent Assay (ELISA) Tests

Hippocampal IGF1 concentration was measured using a Mouse IGF1 ELISA Kit (Elabscience Biotechnology Co., Ltd., Wuhan, China). Hippocampal tissues were homogenized in an appropriate volume of RIPA buffer supplemented with a phosphatase and protease inhibitor cocktail, then transferred to tubes and centrifuged at 5000 × g for 10 min at 4°C. The supernatant was collected for subsequent analysis. All ELISA procedures were performed according to the manufacturer's instructions. Absorbance was measured at 450 nm using a TECAN Spark microplate reader (Tecan Trading AG, Switzerland), and IGF1 concentrations were determined from a standard curve. Total protein concentration of the supernatant was quantified by BCA assay, and calculated the content of IGF1 in the hippocampus.

### 4.9 Immunohistochemistry and Immunofluorescence Staining

The expression of IGF1 and IGF1R in the hippocampus was detected by an immunohistochemistry kit (MXB biotechnologies, KIT‐9720). Mice were euthanized and perfused with saline, followed by 4% paraformaldehyde. Brains were removed to gradient alcohol dehydrated, and embedded in paraffin. Coronal hippocampal sections were cut at 4 µm, dewaxed with graded ethanol, and subjected to antigen retrieval using Citrate Antigen Retrieval Solution in a microwave oven. After washing with PBS, the sections were incubated with the kit reagents according to the manufacturer's instructions, followed by overnight incubation at 4°C with the following primary antibodies: IGF1 (Abcam, ab9572, 1:50) and IGF1R (Proteintech, 66283‐1‐Ig, 1:800). Sections were then developed with a DAB chromogenic kit, counterstained with hematoxylin for 2 min, and mounted with neutral balsam. IGF1 and IGF1R expression in the hippocampus was examined under a light microscope.

Immunofluorescence staining for IGF1R, GLUT3, and Neuronal Nuclei (NeuN) was performed on hippocampal sections. The tissue preparation, including perfusion, paraffin embedding, dewaxing, and antigen retrieval, was carried out as described above for immunohistochemistry. Sections were blocked with blocking solution at 37°C for 1 h, then incubated overnight at 4°C with the following primary antibodies: IGF1R (Proteintech, 66283‐1‐Ig, 1:1000) or GLUT3 (Abcam, ab191071, 1:1000), together with NeuN (Abcam, ab104224, 1:200). The following day, sections were incubated with goat anti‐mouse Alexa Fluor 594 (Proteintech, RGAM004, 1:500) and goat anti‐rabbit Alexa Fluor 647 (Proteintech, RGAR005, 1:500) secondary antibodies, then mounted with Antifade Mounting Medium containing DAPI. The IGF1R^+^/NeuN^+^ and GLUT3^+^/NeuN^+^ cells in the hippocampus were observed by laser scanning confocal microscopy. The subcellular distribution of GLUT3 was further quantified using a custom Cell Profiler pipeline. Briefly, individual cellular compartments, including the cytoplasm and plasma membrane, were segmented, and fluorescence intensity features were extracted to calculate the membrane‐to‐cytoplasm fluorescence intensity ratio of GLUT3 [67].

### 4.10 Thioflavin S Staining

Aβ was visualized by Thioflavin S staining. A 0.3% Thioflavin S solution was prepared by dissolving the dye in 50% ethanol. Sections were incubated in the staining solution for 8 min in the dark, washed three times with 50% ethanol and once with PBS, then mounted with Antifade Mounting Medium containing DAPI. Images were acquired by laser scanning confocal microscopy.

### 4.11 Adenosine Triphosphate (ATP) and Tricarboxylic Acid Cycle Associated Enzymes Content Determination

ATP content was measured using an Enhanced ATP Assay Kit (Beyonctime Biotechnology, Shanghai, China, S0027). Hippocampal proteins were extracted in ATP lysis buffer, and a portion of the lysate was reserved for protein quantification by BCA assay. Subsequently, 20 µl of supernatant was combined with 100 µl of ATP assay reagent in a 96‐well black microplate and incubated at room temperature for 2 s. Luminescence was measured using a microplate luminometer, and ATP levels were expressed as nmol/mg protein.

The activities of tricarboxylic acid (TCA) cycle‐associated enzymes in hippocampal tissue were determined using colorimetric assay kits: Citrate Synthase Activity Assay Kit (Elabscience, E‐BC‐K178‐M), NAD‐Isocitrate Dehydrogenase Activity Assay Kit (Elabscience, E‐BC‐K651‐M), α‐Ketoglutarate Dehydrogenase Activity Assay Kit (Elabscience, E‐BC‐K083‐M), and NAD‐Malate Dehydrogenase Activity Assay Kit (Elabscience, E‐BC‐K561‐M). Protein concentration of each sample was determined prior to the assay, and all procedures were performed according to the manufacturer's instructions. Enzyme activities were expressed as nmol/mg protein.

### 4.12 Nissl Staining

Brain sections were stained with Nissl staining solution (Beijing Solarbio Science & Technology Co., Ltd.) at 56°C for 30 min, then washed with pure water twice for 10 s each time. Sections were differentiated in 95% ethanol for 5 s, then mounted with neutral balsam. Results were examined under a light microscope.

### 4.13 Golgi Staining

Golgi staining was performed using a Golgi Staining Kit (Fdneurotech, PK401A). Following euthanasia, brains were extracted and immersed in impregnation solution, then stored in the dark at room temperature for two weeks. Tissue blocks were gently agitated twice per week for a few seconds. Brain tissue was then embedded in optimal cutting temperature (OCT) compound and sectioned at 100 µm using a frozen microtome. Sections were allowed to air‐dry in the dark for 48 h, then washed twice with water for 4 min each. Sections were subsequently immersed in staining solution for 10 min, prepared by mixing solutions D, E, and double‐distilled water at a 1:1:2 ratio. Following staining, sections were dehydrated through a graded ethanol series of 50%, 75%, and 90% for 4 min each, followed by four changes of absolute ethanol for 4 min each. Sections were then cleared in xylene for three changes of 4 min each and mounted with neutral balsam. Slides were stored in the dark until imaging. Dendritic morphology in the hippocampus was examined at ×630 magnification.

### 4.14 Statistical Analysis

All data were analyzed using SPSS 24.0 and presented as mean ± standard deviation (x̄ ± SD). For comparisons among multiple groups, one‐way ANOVA was applied, with post‐hoc testing performed using the LSD method for homogeneous variances or the Dunnett's T3 method for heterogeneous variances. Non‐normally distributed data were compared across groups using the Kruskal‐Wallis H test. The exact *p*‐values were reported for all comparisons.

## Conflicts of Interest

The authors declare no conflicts of interest.

## Data Availability

The data that support the findings of this study are available from the corresponding author upon reasonable request.
